# C-type lectins and extracellular vesicles in virus-induced NETosis

**DOI:** 10.1186/s12929-021-00741-7

**Published:** 2021-06-11

**Authors:** Pei-Shan Sung, Shie-Liang Hsieh

**Affiliations:** 1 Genomics Research Center, Academia Sinica, 128, Academia Road, Sec. 2, Nankang District, Taipei, 115 Taiwan; 2 Institute of Clinical Medicine, National Yang Ming Chiao Tung University, Taipei, Taiwan; 3 Department of Medical Research, Taipei Veterans General Hospital, Taipei, Taiwan; 4 Institute for Cancer Biology and Drug Discovery, Taipei Medical University, Taipei, Taiwan; 5 Institute of Immunology, College of Medicine, National Taiwan University, Taipei, Taiwan

**Keywords:** COVID-19, SARS-CoV-2, Dengue virus (DV), Platelet, C-type lectin receptor (CLR), CLEC2, CLEC5A, TLR, Extracellular vesicle (EV), Microvesicle (MV), Exosome (EXO)

## Abstract

Dysregulated formation of neutrophil extracellular traps (NETs) is observed in acute viral infections. Moreover, NETs contribute to the pathogenesis of acute viral infections, including those caused by the dengue virus (DV) and severe acute respiratory syndrome coronavirus-2 (SARS-CoV-2). Furthermore, excessive NET formation (NETosis) is associated with disease severity in patients suffering from SARS-CoV-2-induced multiple organ injuries. Dendritic cell-specific intercellular adhesion molecule-3-grabbing non-integrin (DC-SIGN) and other members of C-type lectin family (L-SIGN, LSECtin, CLEC10A) have been reported to interact with viral glycans to facilitate virus spreading and exacerbates inflammatory reactions. Moreover, spleen tyrosine kinase (Syk)-coupled C-type lectin member 5A (CLEC5A) has been shown as the pattern recognition receptor for members of flaviviruses, and is responsible for DV-induced cytokine storm and Japanese encephalomyelitis virus (JEV)-induced neuronal inflammation. Moreover, DV activates platelets via CLEC2 to release extracellular vesicles (EVs), including microvesicles (MVs) and exosomes (EXOs). The DV-activated EXOs (DV-EXOs) and MVs (DV-MVs) stimulate CLEC5A and Toll-like receptor 2 (TLR2), respectively, to enhance NET formation and inflammatory reactions. Thus, EVs from virus-activated platelets (PLT-EVs) are potent endogenous danger signals, and blockade of C-type lectins is a promising strategy to attenuate virus-induced NETosis and intravascular coagulopathy.

## Background

Neutrophils express abundant Toll-like receptors (TLRs) that recognize pathogen-associated molecular patterns in bacteria, viruses, and other microbes [1]. Moreover, neutrophils also express abundant spleen tyrosine kinase-coupled C-type lectin receptors (Syk-CLRs) to serve as pattern recognition receptors (PRRs) to recognize beta-glucans on fungi (Dectin-1 and Dectin-2) [2] and peptidoglycans on *Listeria* (spleen tyrosine kinase (Syk)-coupled C-type lectin member 5A, CLEC5A) [3]. Neutrophil-mediated immunity is via phagocytosis, release of free radicals, and other mediators to kill microbes [4]. Recently, neutrophils are found to prevent bacteria spreading via the formation of web-like structures, known as neutrophil extracellular traps (NETs) [5]. In contrast to bacteria and fungi, viruses exist on a nanometer scale and enter cells via membrane fusion. Therefore, hosts rely on humoral immunity (antibodies) to neutralize viruses as well as cell-mediated immunity to kill virus-infected cells to prevent virus invasion. From this viewpoint, neutrophils seem dispensable, or only play a minor role, in anti-viral immunity. Thus, the significance of virus-induced NET formation in host defense is still controversial, and the molecular mechanism of virus-induced NETosis needs to be further investigated. In our recent review article, we addressed the detrimental roles of two Syk-CLRs (CLEC2 and CLEC5A) in acute viral infections [6, 7], and blockade of Syk-CLRs seems promising to attenuate virus-induced NETosis and injuries [8]. We would like to further address the critical roles of the Syk-CLR (CLEC2) and non-Syk-CLR (dendritic cell-specific intercellular adhesion molecule-3-grabbing non-integrin, DC-SIGN) in the release of extracellular vesicles (EVs) from virus-activated platelets (PLT-EVs), and discuss the potential roles of CLEC5A and Toll-like receptor 2 (TLR2) in PLT-EVs-mediated NETosis and tissue injuries in acute viral infections.

## Virus-induced NET formation

Neutrophils comprise more than 50% of leukocytes in human peripheral blood and are the most abundant effector cells in human innate immunity. Neutrophils produce NETs, which comprise decondensed chromatin, histones, subsets of granules and cytoplasmic proteins that ensnare a variety of microbes [5]. NET release primarily occurs through a cell death process termed NETosis, which is characterized by the disassembly of the nuclear envelope, decondensation of nuclear chromatin into the cytoplasm, and mixture of the nuclear, cytoplasmic, and granular components in the cytoplasm of intact cells. At 3–8 h post neutrophil activation and subsequent cell death, NETs gradually expand into the extracellular space. In contrast to classical NETosis, a small population of neutrophils release NETs within minutes after exposure to *Staphylococcus aureus* in the absence of cell death (i.e., non-lytic NETosis) [9].

Decondensation of chromatin is one of the most important features of NETosis, and the process is dependent on the activation of protein-arginine deiminase type 4 (PAD4), an enzyme that citrullinates arginine residues of histones in nucleus [10]. The degree and specificity of citrullination seems depending on the activation of different PKC isoforms after engagement of neutrophils with various danger signals. PAD4 deficient neutrophils fail to produce NETs upon stimulation with chemokine or bacteria [10], while PAD4 deficient mice are more susceptible to *Streptococcus pyogenes* infection [11]. This observation demonstrates the critical role of NET formation in anti-bacterial infection. In contrast to the beneficial effects of NETs in anti-bacterial infection, excessive NET formation is detrimental in other microbial infections. NETs directly kill epithelial and endothelial cells, and excessive NETs damage pulmonary epithelium in fungal infection [11]. Moreover, excessive NETs in blood vessels provide scaffolds to promote deep vein thrombosis [9]. This observation suggests that excessive NETs not only damage vascular endothelial cells, but also promote intravascular coagulation and blood vessel occlusion.

The first report of virus-induced NET formation was observed in a macrophage-depleted mouse model infected with influenza A virus (IAV) H1N1 strain PR8 [12]. The authors found excessive neutrophil infiltration with extensive NET formation at the terminal bronchioles of mice after H1N1 infection [12]. Mechanically, the authors showed direct induction of NETosis by incubating neutrophils with IAV-infected LA-4 epithelial cells (multiplicity of infection/MOI = 20) for 5 h, followed by incubation with neutrophils for 150 min [12]. This study indicates that excessive NET formation after H1N1 infection contributes to acute lung injury and acute respiratory distress syndrome (ARDS). However, whether virus per se can activate neutrophils and induce robust NET formation is not addressed in this study. Another study demonstrated that intranasal inoculation of IAV induced NET formation and alveolar damage, which was further enhanced by secondary bacterial infection [13]. Later findings also revealed that a high level of NETs correlated with poor prognosis in severe influenza infections [14]. These observations suggest that excessive NET formation may contribute to the pathogenesis of acute lung injury in IAV-induced pneumonia. However, the latter two studies did not demonstrate whether incubation of neutrophils and IAV could induce NET formation in vitro.

Saitoh et al*.* later demonstrated that incubating pseudotyped human immunodeficiency virus-1 (HIV-1) with human neutrophils for 24 h induced the formation of multilobulated structures comprising DNA-based fibers, suggesting that HIV-1 induced NET formation in vitro. To understand how HIV-1 triggered NET formation, cells were treated with bafilomycin A1 to inhibit H^+^-ATPase, which is essential for endosomal TLR activation [15]. Inhibition of HIV-1-induced NET formation by bafilomycin A1 led the authors to conclude that the activation of endosomal TLR7 and TLR8 mediated HIV-1-induced NET formation. The authors further demonstrated that NETs captured HIV-1 and promoted HIV-1 elimination, indicating NET formation is beneficial to host by inhibiting viral infection and spreading. Nevertheless, the authors did not demonstrate that HIV-1 could induce NET formation in vivo. Because NET formation usually occurs at 90 min after incubation with *S. aureus*, immune complexes, or phorbol myristate acetate (PMA) [16], the extremely slow kinetics (24 h) of HIV-1-induced NET formation suggests that HIV-1 is a very weak inducer of NET formation in vitro. Intriguingly, the authors also demonstrated that HIV-1 induced DC-SIGN-dependent IL-10 production to counteract NET formation. Thus, whether HIV-1-induced NET formation is beneficial to host needs to be further investigated.

In contrast to the slow kinetics of HIV-1-induced NET formation, human respiratory syncytial virus (RSV) and hantaan virus (HTNV) induce NET formation within 3–8 h in vitro [17, 18], while myxoma virus (MYXV) induces NET formation within 8 h after injection to mice [19]. Cortjens et al*.* demonstrated that a 3-h incubation of human RSV with neutrophils induces mild non-cytolytic NETs comprising loose web-like DNA networks overlaid with elastase and citrullinated histone in vitro [17]. Interestingly, stronger NET formation was observed in the airways and lungs of children after RSV infection. Moreover, the extent of NET formation correlates with the severity of lower respiratory tract disease after RSV infection. Therefore, the authors concluded that exaggerated NET formation contributed to airway obstruction, and played a detrimental role in RSV infection. Furthermore, Sung et al*.* demonstrated that dengue virus (DV) induced NET formation via inducing platelet-derived EVs, and blockade of NET formation reduced DV-induced hemorrhagic shock [20]. Recently, NET formation is shown as a disease severity marker in COVID-19 patients [21], and contributes to the pathogenesis of severe acute respiratory syndrome coronavirus-2 (SARS-CoV-2) infection, including acute lung injury [22, 23], neuroinflammation [24], and vascular occlusion [25–27]. As SARS-CoV-2 triggers NETs to mediate COVID-19 pathology [28], it is crucial to understand the molecular mechanism of SARS-CoV-2-induced NET formation for the development of better therapeutic strategy in the future [29]. The kinetics of NET formation among various viruses are summarized in Table 1 [12, 13, 15, 17–21, 30].

**Table 1:** Comprehensive list of virus-induced NET formation in vitro and in vivo

Virus		In Vitro					In Vivo					
		Species	Origin of neutrophils	Induction of NETs	Induction time (hour)	Detection of NET structure	Species	Induction of NETs	Induction time	Occurred organs	Detection of NET structure	Ref
Influenza A virus (IAV)	H1N1 (PR8)	Mouse, BALB/c	BALF from uninfected BALB/c mice	Cocultured with influenza- primed LA-4 cells	2.5	IF^a^	Mouse, BALB/c	500 PFU (i.n.)	6 days post- infection	Lung	H&E	12
											IF^b^	
		Mouse, C57BL/6	BM-derived neutrophils	BAFL from infected mice	0.5	IF^c^	Mouse, C57BL/6	500 PFU (i.n.)	5 days post- infection	Lung	IF^c^	13
	H3N2 (Phil82)	Human	Peripheral blood	MOI = 50	3	SYTOX Green	N/A	N/A	N/A	N/A	N/A	30
Human immunodeficiency virus-1 (HIV-1)		Human	Peripheral blood	Pseudotyped HIV-1 (1–6 ng/ml)	24	Hoechst	N/A	N/A	N/A	N/A	N/A	15
Myxoma virus (MYXV)		N/A	N/A	N/A	N/A	N/A	C57BL/6 mice	1 × 10^7^ PFU (i.v.)	8 h post infection	Liver	Intravital images*	19
Respiratory syncytial virus (RSV)		Human	Peripheral blood	MOI = 0.0005–0.005	3	IF^d^ Quant-iT dsDNA kit	N/A	N/A	N/A	N/A	N/A	17
Hantaan virus (HTNV)		Human	Peripheral blood	MOI = 1	6	SYTOX Green	Human , infected patients	N/A	N/A	Kidney and serum	IF^e^ ELSIA^a^	18
Dengue virus (DV) (serotype 2)												
Mouse C57BL/6		BM-derived neutrophils		8								
PL046		Human	Peripheral blood	MOI = 5	3	IF^f^	*stat1*^*−/−*^ mice	2 × 10^5^ PFU (i.c./ i.p.)	5 days post infection	Spleen	IF^g^	20
NGC-N		Mouse C67BL/6	BM-derived neutrophils	MOI = 3								
Severe acute respiratory syndrome coronavirus-2 (SARS-CoV-2)		N/A	N/A	N/A	N/A	N/A	Human, infected patients	N/A	N/A	serum	ELISA^b^	21

IF^a^ = MPO, phalloidin, DNA; IF^b^ = histone H2B and DNA; IF^c^ = histone H2B, MPO, and DNA; IF^d^ = MPO, elastase, and DNA; IF^e^ = histone, elastase,and DNA; IF^f^ = histone H1, MPO, and DNA; IF^g^ = Cit-Histone H3, MPO,and DNA. ELISA^a^ = dsDNA/histone complex; ELISA^b^ = cell-free DNA, MPO-DNA, and Cit-H3. Intravital images* = histone H2Ax and elastase

*NGC-N* New Guinea C-N, *IF* immunofluorescence staining, *MPO* myeloperoxidase, *BALF* bronchoalveolar lavage fluid, *MOI* multiplicity of infection, *N/A* not available, *BM*  bone marrow, *PFU* plaque-forming unit, *i.n* intranasal injection, *i.v* intraveseel injection, *i.c* intracerebral injection, *i.p* intraperitoneal injection

## Activated platelets in NET formation

While virus alone is a weak inducer of NET formation in vitro, viral infection can induce substantial NET formation in vivo. This observation suggests that other cells may participate in virus-induced NET formation. Platelets are derived from myeloid precursors and are regarded as key players in hemostasis and thrombosis. Similar to other myeloid cells, platelets express abundant pattern recognition receptors, including TLRs and Syk-CLRs. Several reports suggest that activated platelets can induce NET formation in various model systems.

### Thrombin-activated platelets promote NET formation

Although thrombin receptor-activating peptide (TRAP, a PAR-1 agonist) has no effect on NET formation, incubation of TRAP-activated platelet with neutrophils induces robust NETs in vitro [31]. In addition, excessive NETs contribute to transfusion-related acute lung injury (TRALI), and inhibition of platelet activation by aspirin or glycoprotein IIb/IIIa inhibitors decreases NET formation and attenuates lung injury [31]. Taken together, these observations indicate that activated platelet play a critical role in TRALI. Because TRAP-1 activated *Hmgb1*^−/−^ platelets are far less potent inducers of NET formation compared to wild-type platelets, NET formation by thrombin-activated platelets is likely mediated by high mobility group box 1 (HMGB1) [32]. Intriguingly, incubation of HMGB1 protein with neutrophils does not induce NET formation in vitro, suggesting HMGB1 is just one of the components of platelet-released NET inducer.

### Microbe-activated platelets promote NET formation

Activation of platelets during infections is not only able to enhance leukocyte functions and NET formation via direct and indirect interactions, but also contributes to pathogen-induced tissue injury [33]. Several bacteria have been shown to interact and activate platelets via glycoprotein (GP)IIb-IIIa, GPIbα, FcγRIIa, complement receptors, and TLRs [34]. In addition, lipopolysaccharide (LPS)-activated platelets interact with neutrophils to induce strong NET formation both in vitro and in vivo [35]. However, it is still unclear how virus-activated platelets induce NET formation.

Influenza virus infection frequently causes excessive neutrophil-platelet aggregates and NET formation in lung, thereby contributes to severe lung pathological changes [36]. It has been demonstrated that IAV activates platelets via TLR7 to release complement component 3 (C3) to induce NET formation in a mouse model [37]. Addition of C3 (30 ng/ml) to neutrophils induces NET formation in vitro, suggesting TLR7 plays a critical role in HIV-1-induced platelet aggregation, C3 releasee, and NET formation [37]. However, considering the high level of C3 (0.8–1.6 mg/ml) in human serum, the pathological roles of platelet-derived C3 (30 ng/ml) in IAV-induced NET formation need to be further validated.

## Platelets and EVs

In addition to complement, DV can activate platelets to release EVs (also known as microparticles) [38] to promote DV-induced inflammatory reactions and NET formation significantly [20]. While all cell types can produce EVs, platelets are the major source of circulating EVs in sera [39]. EVs are heterogeneous groups of cell-derived membranous structures, and the typical size range of EVs is between 50 and 500 nm. Based on the origins and size, EVs are divided into exosomes (EXOs) and microvesicles (MVs). EXOs (50–150 nm) are originated from endosomes, while MVs (average 50–500 nm, up to 1–10 μm) are derived from plasma membrane [40]. In addition to lipid and proteins in the lipid-bilayer membrane, EVs contain various kinds of macromolecules, including enzymes, beta-catenin, G proteins, 14-3-3, chaperons, microRNA, non-coding RNA, mRNA, and DNA. Once released from cells, EVs deliver these molecules to target cells via membrane fusion or internalization after binding membrane receptors. Furthermore, EVs bind to integrins, proteoglycans, lipid-binding proteins, and phosphatidylserine receptor TIM4 to initiate intracellular signaling cascades. Therefore, EVs were considered as an alternative mechanism for intercellular communications [41] and activation of intracellular signaling cascades.

### EVs from virus-infected cells

EVs released from virus-infected cells contain virus- and host-derived factors to facilitate virus spreading. For example, several enveloped viruses, such as HIV-1 and hepatitis viruses B, C (HBV, HCV) utilize the endosomal sorting complexes required for transport (ESCRTs) to pack viral components to facilitate virus transmission. Moreover, EVs can spread viral docking receptors to promote viral infectivity or inhibit anti-viral responses [42–44]. These observations suggest that EVs from virus-infected cells play critical roles in intercellular communications, and may help viruses to escape from host immunosurveillance and promote transmission by hiding their genome in EVs.

### EVs from virus-activated platelets

While EVs from platelet-rich plasma (PRP) promotes cell proliferation and tumor progression [45], EVs from complement-activated platelets posse procoagulant activity [46], suggesting complements play detrimental role in NETs-mediated effects Further studies indicated PLT-EVs also participate in intracellular communication, angiogenesis, tumor progression, inflammation, immunoregulation, and cellular prion protein transport [45].

In the past, research attention focused on EV-mediated delivery of proteins and RNAs to dendritic cells, and investigated their effects to enhance antigen presentation and modulate cell activity [47]. However, PLT-EVs also carry proinflammatory cytokines and contribute to fibrin deposition and joint inflammation in rheumatoid arthritis patients [48]. Recent studies further demonstrate that virus-activated platelets release EVs to enhance NET formation via activation of C-type lectins. [49–51].

## C-type lectins and NET formation

Viruses not only utilize various types of specific entry receptors to invade cells, but also engage with target cells via glycan-lectin interactions. The conventional carbohydrate-recognition domain (CRD) with EPN (Glu-Pro-Asn) tripeptide motif binds mannose (Man), N-acetylglucosamine (GlcNAc), glucose (Glc), and L-fucose (Fuc), while CRDs with QPD (Glu-Pro-Asp) tripeptide motif binds galactose (Gal) and N-acetylgalactosamine (GalNAc)[52, 53]. Many reports have shown that viruses can interact with lectins in immune cells via terminal glycans on the virus surface[54–57]. Viral glycan-lectin interaction is not only involved in virus entry, but also plays a very important role in activating immune cells and triggering inflammatory reactions. Among members of the human C-type lectin family, DC-SIGN, L-SIGN, LSECtin, CLEC10A [58], as well as the Syk-coupled C-type lectins CLEC5A and CLEC2 [20], have been shown to play critical roles in virus-induced inflammation and NETosis.

### C-type lectins in platelets

Among the C-type lectin family, DC-SIGN and the CLEC2 are highly expressed in platelets. While DC-SIGN is expressed in platelets and most of the myeloid cells [59], CLEC2 is expressed specifically in human platelets and megakaryocytes [60, 61].

#### DC-SIGN

DC-SIGN is the best-studied EPN-containing C-type lectin, and is highly expressed in myeloid cells, including macrophages, dendritic cells, neutrophils, and platelets [62]. Previous reports show that the specific interactions between DC-SIGN and viruses depend on the viral glycans and the CRD of DC-SIGN [63–73]. DC-SIGN forms a homo-tetramer and interacts with several glycans, including high-mannose, Lewis a/b/x/y, and fucosyl biantennary N-glycans[74]. DC-SIGN is also reported to interact with several viruses to facilitate virus spread, infection, and stimulation of inflammatory reactions. For example, HIV-1 binds platelets via DC-SIGN to facilitate virus spread [60, 75, 76]. Moreover, DC-SIGN also interacts with the DV [77], West Nile virus (WNV) [66], Japanese encephalomyelitis virus (JEV) [78], Lassa virus (LVs) [79], measles virus (MVs) [80], H5N1 IAV [81], and feline coronaviruses[82] to facilitate virus entry into dendritic cells. Recent studies further demonstrate that DC-SIGN facilitates the entry of the New World arenavirus, Junin virus [83], and Rift Valley Fever (RVF) virus [63] into host cells, and act as an attachment-promoting receptor to boost Ebola virus entry into B cell lines [83–85]. Moreover, HCV interacts with DC-SIGN to escape lysosomal degradation [86]. Because the cytoplasmic domain of DC-SIGN does not contain motifs for signal transduction, it is unlikely that viruses can activate platelets directly via DC-SIGN.

#### CLEC2

CLEC2 is a Syk-CLR that is specifically expressed in human platelets. Human CLEC2 encodes a 229–amino acid type II transmembrane protein, comprising a C-type lectin domain in the C-terminus, and a single YxxL motif (hemITAM) in the intracellular domain located at the N-terminus [60, 61]. In contrast to conventional C-type lectins, CLEC2 does not contain an EPN or a QPD tripeptide motif in its CRD domain. Nevertheless, CLEC2 is reported to bind various glycans, including sulfated N-acetyl lactosamine (LacNAc), sulfated lactose (Lac), poly 2–8-linked N-acetyl neuraminic acid (NeuAc), high-mannose, and sialyl-LacNAc/Lac/Lewis a[74, 87, 88]. A recent study further demonstrates that CLEC2 can bind sulfated polysaccharides fucoidans specifically [89]. While O-linked glycoprotein podoplanin is the only known endogenous ligand of CLEC2, snake venom aggretin (rhodocytin) from *Calloselasma rhodostoma* is shown to induce platelet activation and aggregation via CLEC2 [61, 90]. Studies from CLEC2 knockout mice further suggest that CLEC2 is required for blood/lymphatic vessel separation during embryo development [91, 92]. In addition, CLEC2 is responsible for immunothrombosis in bacterial infections [93, 94], and CLEC2 deficiency increases susceptibility to LPS-induced sepsis [95]. Furthermore, CLEC2 interacts with DC-SIGN to capture HIV-1 and facilitates its dissemination in infected patients [60]. Our recent study demonstrates that DV activates platelets via CLEC2 to release EVs [20], despite very low interaction between CLEC2 and DV [54]. This observation suggests that viruses can be captured by DC-SIGN to facilitate virus binding and activating platelets via CLEC2.

### CLEC2 is critical in virus-induced EV release

A recent study by Sung et al*.* demonstrates that DV activates platelets to upregulate the release of CD62P^+^CD63^+^ EVs, including DV-activated EXOs (DV-EXOs) and MVs (DV-MVs). Addition of anti-CLEC2 mAb abolishes DV-EV release from human platelets, indicating CLEC2 is required for DV-induced EV release. The authors further separate DV-EVs into DV-EXOs and DV-MVs, and find that DV-EXOs-induced NET formation is inhibited by antagonistic anti-CLEC5A mAb, while DV-MVs-induced NET formation is inhibited by anti-TLR2 mAb. This observation suggests that DV-EXOs and DV-MVs activate CLEC5A and TLR2, respectively, to enhance NET formation and proinflammatory cytokines release from macrophages[20]. Thus, EVs from DV-activated platelets can act as endogenous ‘danger signals’ to enhance inflammatory reactions via activation of CLEC5A and TLR2. Furthermore, incubation of DV-EVs with endothelial cells increased permeability changes, whereas blocking the interactions between DV-EVs and CLEC2 not only inhibited NET formation and attenuated systemic permeability change in vivo, but also protected mice from DV-induced lethality dramatically (> 90% protection rate) [20]. Because several viruses have been shown to interact with DC-SIGN, to form multivalent heterocomplex with CLEC5A and mannose receptor (MR), respectively [96], it would be interesting to investigate whether DC-SIGN also associates with CLEC2 to form CLEC2/DC-SIGN heterocomplex in platelets, therefore allows viruses to activate CLEC2 to induce EV release via binding to CLEC2/DC-SIGN heterocomplex.

To identify the protein ligands on DV-EVs, Sung et al*.* harvested EVs from DV- and aggretin (CLEC2 ligand)-activated platelets, and subjected these samples to mass spectrometry analysis [20]. The authors found that cytoskeleton components (vinculin), guanine nucleotide-binding protein (GNG3), tribbles homolog 1 (TRIB1), coagulation factor XIIIa chain (F13A1), and calnexin (CANX, an endoplasmic reticulum chaperone) were upregulated by both aggretin and DV, suggesting that these molecules are under the regulation of CLEC2-mediated signaling. Previously, cytoskeletal F-actin is identified as a ligand for C-type lectin member 9A (CLEC9A)[97], and chaperone HSP70 is shown as a ligand for TLR2 and TLR4[98]; therefore, these upregulated components may be responsible for DV-EVs-induced inflammatory reactions during viral infection. It would be interesting to ask whether the proteins upregulated in DV-MVs and DV-EXOs are also found in platelet-derived EVs after incubation of platelets with other members of flaviviruses (such as JEV and WNV) in the future. Moreover, DV infection may cause modulate glycan synthesis in platelets, thus it would be crucial to compare the glycan profile of EV from resting and DV-activated platelets, thereby identify the potential EV glycan ligands to CLEC5A and TLR2. As circulating platelet-derived EVs are a hallmark of SARS-CoV-2 infection [99], it would be interesting to text whether COVID-19 patients’ EVs can activate platelets via protein and glycan ligands to induce NET formation in the future.

### CLEC5A is a Syk-CLR critical in virus-induced NETosis

Several Syk-CLRs have been identified in neutrophils, including CLEC7A (Dectin-1), CLEC6A (Dectin-2), CLEC4E (Mincle), and CLEC5A (MDL-1)[100]. Among these four Syk-CLRs, CLEC5A is shown to be responsible for *Listeria monocytogenes*- and DV-induced NETosis and macrophage activation [3, 20].

#### CLEC5A

CLEC5A is abundantly expressed in neutrophils, monocytes, macrophages, osteoclasts, microglia, and dendritic cells. CLEC5A serves as a pattern recognition receptor for DV [54, 101, 102], JEV[103], and the IAV [104]. Furthermore, CLEC5A is responsible for DV-induced hemorrhagic fever (DHF) and dengue shock syndrome (DSS), which represent the most severe responses to DV infection and are characterized by plasma leakage due to increased vascular permeability [54]. Injection of anti-CLEC5A monoclonal antibodies (mAb) can reduce mortality (from 100 to 40%–50%) in mice subjected to a lethal-dose DV challenge [54]. Similarly, blockade of CLEC5A prevents JEV-induced permeability changes in the blood–brain barrier, and protects mice from neuroinflammation and mortality[103]. These observations suggest that CLEC5A is a promiscuous pattern recognition receptor to viruses[7], and plays as a pathogenic host factor in flaviviral and influenza virus-induced inflammatory reactions.

Because direct interactions between CLEC5A and DV is weak [105], the beneficial effect of anti-CLEC5A mAb is not only mediated by inhibiting DV-CLEC5A interactions, but also by blocking interactions between CLEC5A and its endogenous danger signals. Even though the nature of CLEC5A endogenous ligands is not been characterized yet, CLEC5A-deficient mice are resistant to collagen-induced autoimmune arthritis [106] and concanavalin A-induced acute hepatitis. [107]. These observations suggest that CLEC5A can recognize endogenous danger signals, and is involved in the pathogenesis of autoimmune diseases.

#### CLEC5A/TLR2 heterocomplex in EVs-induced NETosis and inflammation

To understand whether CLEC5A and TLR2 also contribute to DV-EVs–induced NET formation, Sung et al. incubated DV-EVs with neutrophils pretreated with anti-CLEC5A mAb and anti-TLR2 mAb. While DV-EVs-induced NET formation was partially inhibited by anti-CLEC5A mAb or anti-TLR2 mAb, co-administration of anti-CLEC5A mAb and anti-TLR2 mAb almost completely abolished DV-EVs-induced NETosis and inflammatory reactions[20]. The authors further asked whether blockade of CLEC5A and TLR2 is beneficial to host after DV infection in vivo. To address this question, *stat1*^*−/−*^ mice and *stat1*^*−/−*^* clec5a*^*−/−*^ mice were challenged with lethal dose of DV, followed by anti-TLR2 mAb injection. While blockade of TLR2 alone was ineffective in DV-challenged *stat1*^*−/−*^ mice, anti-TLR2 mAb reduced DV-induced NET formation and increased the survival rate of DV-challenged *stat1*^*−/−*^* clec5a*^*−/−*^ mice from 40 to 90% [20]. This observation suggests that simultaneous blockade of CLEC5A and TLR2 not only reduced NET formation in vivo, but also protected mice from DV-induced hemorrhagic shock and lethality [21]. Thus, DV-EVs are potent NET inducers, and simultaneous blockade of CLEC5A and TLR2 by bi-specific mAbs may be able to abolish virus-induced NET formation, and protect host from virus-induced lethality [8].

It has been shown that engagement of macrophages or neutrophils with *L. monocytogenes* induces colocalization and co-activation of CLEC5A and TLR2 [3]. Co-activation of CLEC5A and TLR2 by *L. monocytogenes* induced p38 kinase and AKT kinase activation, leading to robust NET formation in neutrophils as well as inflammasome (NALP3, NLRC4, AIM2) activation and proinflammatory cytokine (IL-1 beta, TNF, CCL2, IL-17 alpha) production in macrophages [3]. Because DV-EVs can activate CLEC5A and TLR2 to induce NET formation and proinflammatory cytokine release, simultaneous blockade of CLEC5A and TLR2 by a bi-specific mAb may be able to protect host from DV-EVs-induced NETosis and inflammatory reactions during viral infections.

## C-type lectins in the pathogenesis of COVID-19 virus infections

While endosomal TLRs are the most potent pattern recognition receptors to viral nucleic acids and are critical in virus-induced interferon production, members of C-type lectins are shown to exacerbate proinflammatory responses in viral infections [20],[58]. Even though viral and endogenous ligands of most C-type lectins need to be further characterized, EVs from virus-activated platelets may act as common endogenous ligands for members of C-type lectins. Recent studies demonstrate that the spike protein of SARS-CoV-2 contains 22 N-linked glycans, including oligomannose, afucosylated and fucosylated hybrid and complex glycans [108]. Thus, it would be interesting to ask whether SARS-CoV-2 activates Syk-CLRs via viral glycans to induce EV release, NET formation, and proinflammatory reactions.

It is interesting to note that EV serum level increased in COVID-19 patients, and correlates with clinical symptoms and lethality [99, 109, 110]. It has been shown that platelets and neutrophils were highly activated, and dysregulated immunothrombosis associated with respiratory failure and coagulopathy was observed in COVID-19 patients. Moreover, incubation of platelet-rich plasma from COVID-19 patients with neutrophils from healthy donor induced robust NET formation [111]. Furthermore, elevated levels of cell-free DNA, myeloperoxidase, and citrullinated histone are noted in the sera of COVID-19 patients, and serum NET amounts correlated with disease severity [112, 113]. These observations suggest that SARS-CoV-2 may activate platelets to release EVs, thereby induce NET formation and cause thrombosis in COVID-19 patients. This speculation is in accord with the increased NET formation in acute lung injury (ALI) caused by SARS-CoV-2 infection [21].

It is also interesting to note that severe pulmonary inflammation in COVID-19 patients is associated with thrombotic complications, such as microangiopathy and pulmonary embolism [114, 115]. As virus-activated platelets form aggregation with neutrophils, platelet-specific CLEC2 may contribute a significant role in SARS-CoV-2-induced immunothrombosis. It has been shown that lung is responsible for 50% of platelet biogenesis or 10 million platelets per hour [116], thus SARS-CoV-2 may activate platelets in lung to release EVs, thereby contributes to ARDS and intravascular coagulopathy. It would be very interesting to ask whether SARS-CoV-2 also activates platelets via CLEC2 to release EVs, and whether blockade of CLEC2 is beneficial to COVID-19 patients in the future.

## Concluding remarks and perspectives

**Fig. 1:**
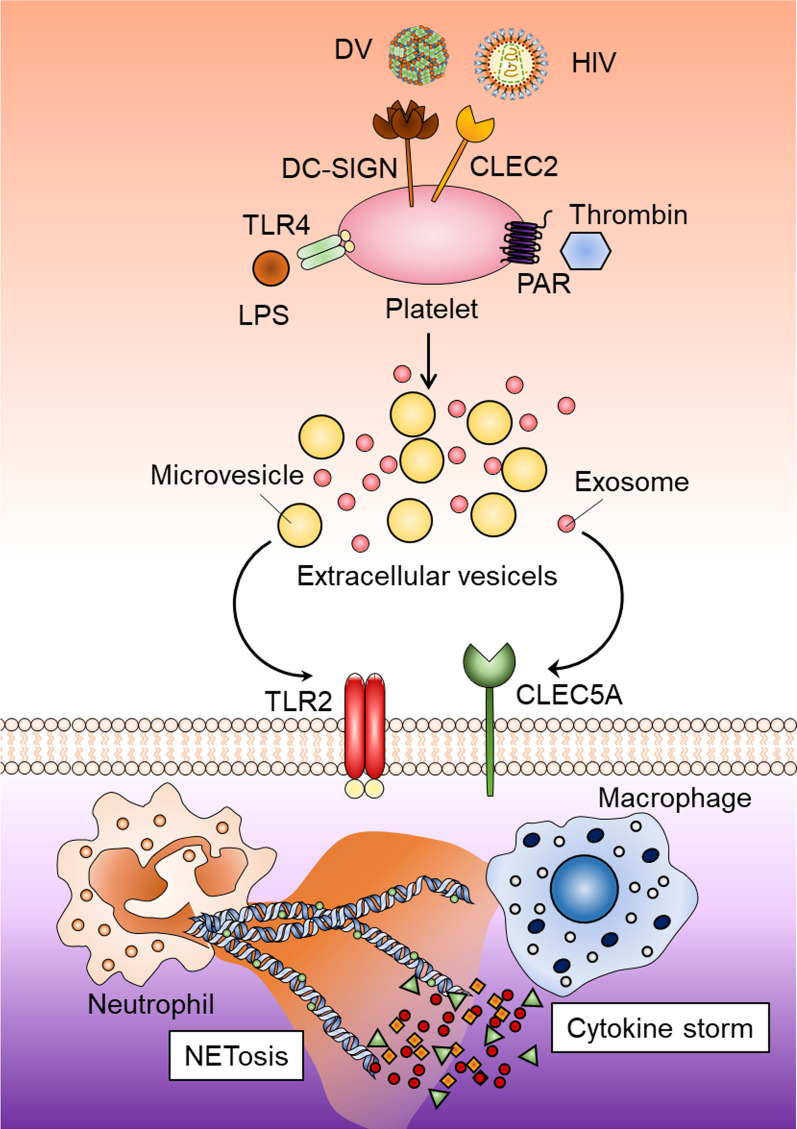
Platelets play a central role in virus-induced NET formation and proinflammatory cytokine release. Dengue virus (DV) and immunodeficiency virus type I (HIV-1) interact with DC-SIGN and CLEC2. While DV, LPS, and thrombin can activate platelets to release extracellular vesicles, including exosomes and microvesicles, it is still unclear whether HIV-1 can activate platelets to release EVs. DC-SIGN may associate with CLEC2 to form DC-SIGN/CLEC2 heterocomplex in platelets, thus facilitate platelets to capture various viruses to activate platelets via CLEC2. Platelet-derived exosomes and microvesicles further activate CLEC5A and TLR2, respectively, to enhance DV-induced NET formation and proinflammatory cytokine release. Thus, EVs from activated platelets may serve as common endogenous danger signals to induce NET formation and inflammatory reactions in various microbial infections

EVs have been implicated in regulation of infectious and autoimmune diseases, but the underlying molecular mechanisms are still unclear. We have demonstrated that platelets secrete EVs after incubation with DV, LPS, and thrombin. Interestingly, all the (PLT-EVs can activate CLEC5A/TLR2 heterocomplex to enhance NET formation and induce the release of IL-1β and other proinflammatory cytokines from macrophages[20] (Fig. 1). This observation suggests the EVs can act as endogenous danger signals to stimulate inflammatory reactions via activating CLEC5A/TLR2 heterocomplex, and blockade of CLEC5A/TLR2-mediated signaling would be able to reduce PLT-EVs-induced inflammatory reactions. It will be very interesting to investigate whether blockade of CLEC5A and TLR2 simultaneously is able to abolish virus-induced inflammatory reactions and inhibit intravascular coagulopathy in COVID-19 patients in the future [6, 20, 29, 50].

## Data Availability

Not applicable.

## Abbreviations

DC-SIGN: Dendritic cell-specific intercellular adhesion molecule-3-grabbing non-integrin
DV: Dengue virus
DV-EXOs: DV-activated EXOs
DV-MVs: DV-activated MVs
EXOs: Exosomes
EVs: Extracellular vesicles
PLT-EVs: EVs from virus-activated platelets
NETs: Neutrophil extracellular traps
JEV: Japanese encephalomyelitis virus
MR: Mannose receptor
MVs: Microvesicles
LacNAc: N-acetyl lactosamine
NeuAc: N-acetyl neuraminc acid
NETosis: NET formation
SARS-CoV-2: Severe acute respiratory syndrome coronavirus-2
CLEC5A: Spleen tyrosine kinase (Syk)-coupled C-type lectin member 5A
CLEC2: Spleen tyrosine kinase (Syk)-coupled C-type lectin member 5A
Syk-CLRs: Spleen tyrosine kinase-coupled C-type lectin receptors
TLR2: Toll-like receptor 2
